# The effect of reward on orienting and reorienting in exogenous cuing

**DOI:** 10.3758/s13415-014-0278-7

**Published:** 2014-03-27

**Authors:** Berno Bucker, Jan Theeuwes

**Affiliations:** Department of Cognitive Psychology, Vrije Universiteit Amsterdam, Van der Boechorststraat 1, 1081 BT Amsterdam, The Netherlands

**Keywords:** Reward, Motivation, Exogenous cuing, Inhibition of return (IOR), Top-down cognitive control

## Abstract

It is thought that reward-induced motivation influences perceptual, attentional, and cognitive control processes to facilitate behavioral performance. In this study, we investigated the effect of reward-induced motivation on exogenous attention orienting and inhibition of return (IOR). Attention was captured by peripheral onset cues that were nonpredictive for the target location. Participants performed a target discrimination task at short (170 ms) and long (960 ms) cue–target stimulus onset asynchronies. Reward-induced motivation was manipulated by exposing participants to low- and high-reward blocks. Typical cue facilitation effects on initial orienting were observed for both the low- and high-reward conditions. However, IOR was found only for the high-reward condition. This indicates that reward-induced motivation has a clear effect on reorienting and inhibitory processes following the initial capture of attention, but not on initial exogenous orienting that is considered to be exclusively automatic and stimulus-driven. We suggest that initial orienting is completely data-driven, not affected by top-down motivational processes, while reorienting and the accompanying IOR effect involve motivational top-down processes. To support this, we showed that reward-induced motivational processes and top-down control processes co-act in order to improve behavioral performance: High-reward-induced motivation caused an increase in top-down cognitive control, as signified by posterror slowing. Moreover, we show that personality trait propensity to reward-driven behavior (BAS-Drive scale) was related to reward-triggered behavioral changes in top-down reorienting, but not to changes in automatic orienting.

Reward and the anticipation of receiving a reward are considered to be central driving forces of all goal-directed behavior and are thought to be the operant concept of motivation (Berridge & Robinson, 1998; Schultz, 1998). There is evidence that reward-induced motivation influences perceptual, attentional, and cognitive control processes to facilitate behavioral performance (Locke & Braver, 2008; Pessoa, 2009). Even though some have argued that reward affects performance independently of attention (see Baldassi & Simoncini, 2011), the majority of studies have shown that reward contingencies have a large impact on the allocation of attention (Anderson, Laurent, & Yantis, 2013; Awh, Belopolsky, & Theeuwes, 2012; Chelazzi, Perlato, Santandrea, & Libera 2013; Hickey, Chelazzi, & Theeuwes, 2010a; Seitz, Kim, & Watanabe, 2009).

It may not be surprising that the expectation of reward has a large effect on those executive processes that are mainly top-down driven. Motivation to perform is known to affect cognitive control and goal-directed behavior. For example, Shen and Chun (2011) showed that offering reward for performance increased flexibility in task switching. Similarly, Jimura, Locke, and Braver (2010) showed that highly reward-sensitive people showed greater improvement in working memory, a task that is considered to involve executive control processes (see also Taylor, 2005). Furthermore, Locke and Braver (2008) showed that the motivational state induced by reward affected the cognitive control strategy of participants. Additionally, their neuroimaging data indicated that reward was associated with a sustained increase of activity in cognitive control regions.

Recently, however, effects of reward have also been implicated in tasks that are less susceptible to reward-induced motivational states. Indeed, several studies have shown that reward contingencies may affect not only top-down control processes, but also perception and selection in itself (see Chelazzi et al., 2013, for a review). For example, Della Libera and Chelazzi (2009) showed that reward affected the allocation of visual attention. This study involved a task that required selective processing of task-relevant information and the concurrent suppression of distracting information. Participants were trained on particular stimulus–reward contingencies between shapes and payment schedules. Five days later, participants performed the same task without obtaining monetary reward. The results showed that reward training had established long-term attentional biases in relation to the specifically rewarded shapes. Crucially, this study showed that a distractor that was associated with a high reward during training was harder to ignore during the test than a distractor that was associated with a low reward during training. This effect was considered to be the result of a learned attentional priority acquired during training, which could not strategically be counteracted during the test phase.

Similarly, Hickey et al. (2010a) came to a similar conclusion. In this study, observers had to search for a unique shape singleton, while an irrelevant color singleton was present (the additional singleton task; Theeuwes, 1991, 1992). Following a correct response, observers received either a low- or a high-magnitude reward. Reward delivery was randomized and not tied to behavioral performance. The colors of all elements randomly switched from trial to trial. The results showed that after receiving a high reward, observers were fast when the target had the same color as on the immediately preceding trial but that they were slow when the colors had switched. For low-reward trials, the pattern reversed: Observers were slow when the target had the same color as on the preceding trial and relatively fast when the colors switched between trials. Low reward resulted in a relative devaluation of features that characterized the target, such that attention was less likely to be deployed to objects characterized by these features on the next trial. Hickey et al. (2010a) concluded that a color associated with high reward on the previous trial automatically captured attention on the current trial, an effect that could not be modulated by top-down strategy (see also Anderson, Laurent, & Yantis, 2011a, 2011b).

The present study was designed to determine whether motivationally driven influences of reward affect exogenous attentional spatial orienting and subsequent suppression of processing, also known as inhibition of return (IOR; see Klein, 2000). Since exogenous orienting and subsequent inhibition is mainly stimulus-driven and automatic (Theeuwes, 2013), it is questionable whether orienting and inhibition are affected by reward-induced motivation. Indeed, even though monetary reward may act as an incentive for improved performance, it is unknown whether processes that are basically completely stimulus-driven are sensitive to reward-induced motivation.

Posner and Cohen (1984) were the first to investigate exogenous attentional orienting. The basic paradigm consisted of three horizontally aligned boxes. Observers had to fixate the central box. During a trial, the outline of one of the peripheral boxes was briefly brightened. This onset served as a peripheral exogenous cue. At a variable stimulus onset asynchrony (SOA), the target was displayed inside one of the boxes, and observers had to detect its presence as quickly as possible by pressing a single key. The results showed that responses were faster when the cue and target appeared at the same location than when the cue and target appeared at different locations, but only when the cue–target SOA was less than 200 ms. With longer SOAs, Posner and Cohen found the reverse: Observers were now faster at detecting targets at the uncued versus the cued location. It was argued that with exogenous cues, the initial capture of attention toward the location of the cue caused early facilitation, which was then followed by inhibition (see also Handy, Jha, & Mangun, 1999). The inhibitory effect due to exogenous spatial orienting was named *inhibition of return* (IOR). IOR is typically found only when attention is exogenously captured by an event (in this case, an abrupt onset). No IOR is found following endogenous spatial orienting (see also Schreij, Theeuwes, & Olivers, 2010; Theeuwes & Chen, 2005; Theeuwes & Godijn, 2002). In studies investigating exogenous orienting, it is crucial that the cue does not predict the location of the upcoming target. If the cue predicts where the target is going to appear, one cannot speak of exogenous, stimulus-driven, bottom-up capture, since observers will use the cue to direct their attention.

It is, however, also possible to direct spatial attention to a location in space “at will.” For example, people are able to direct their attention to a nonfixated location in space. In Posner, Nissen, and Ogden (1978), observers received a central symbolic cue (e.g., an arrow) that pointed to the location of the upcoming target with 80 % validity. This implies that on 80 % of trials, the centrally presented arrow indicated the location where the target was about to appear. On 20 % of the trials, the target appeared at the “invalid” location (i.e., at the location opposite to that indicated by the arrow). Typically, observers were faster and more accurate when the target appeared at the cued location, as compared with the uncued location. It is generally assumed that in response to cues with predictive value, observers endogenously shift their focus of attention to the indicated location.

A study that explicitly addressed whether reward-induced motivation affects exogenous spatial orienting was conducted by Engelmann and Pessoa (2007). In this study, a peripheral onset cue was used that validly indicated the target location on 70 % of the trials. In different blocks, participants were exposed to different reward–punishment contingencies (gaining or losing monetary reward). The results showed that performance (i.e., detection sensitivity) improved as a function of incentive value, for both valid and invalid trials. In other words, there was an overall improvement in performance that did not depend on whether the cue was valid or invalid. The authors concluded that “elevated motivation leads to improved efficiency in orienting and reorienting of exogenous spatial attention” (Engelmann & Pessoa, 2007, p. 668). Even though it is clear that the reward-induced incentive value had an effect on overall performance, it is not immediately clear whether these findings permit the conclusion that reward *“improved the efficiency in orienting and reorienting*” (Engelmann & Pessoa, 2007, p. 668). Indeed, if reward, for example, had affected orienting toward the validly cued location, one would expect that with increased incentive value, sensitivity in detecting a target at the valid location would have improved. Since there was an overall improvement in performance that did not depend on cue validity, it is in fact unlikely that reward affected the orienting of attention. Instead, it seems more likely that reward incentive, which was delivered in blocks, had a general alerting effect affecting overall detection accuracy, but not necessarily the orienting of spatial attention. Another aspect of the design of Engelmann and Pessoa is problematic, since they used cues that predicted one of two target locations with a validity of 70 % (which was not at chance level—i.e., 50 %). Hence, it is hard to argue that this study addressed the orienting following exogenous cuing, since the cues were predictive of the target location.

Small et al. (2005) also examined attentional orienting, but in an endogenous Posner cuing task (in which a cue [i.e., an arrow] was provided at fixation that indicated the correct location of the upcoming target on 80 % of the trials). In such endogenous cuing tasks, orienting toward the likely target location is very much top-down in origin, since it depends on the effort invested by the participant. In this specific experiment, rewarded-induced motivation was manipulated in separate blocks: win money, lose money, or neither win nor lose money. The results showed that incentives improved detection performance for valid and invalid trials, on which a central arrow was used to direct attention. On neutral trials, on which no arrow was provided, there was no significant modulation of performance. Notably, however, consistent with the exogenous cuing as investigated by Engelmann and Pessoa (2007), incentive scheme did not interact with cue validity, indicating that incentives had a general effect on performance, instead of improving attentional orienting itself. As outlined before, if reward-induced motivation affects the actual orienting of attention, one would expect larger validity effects, because observers would be more motivated to direct attention to the cued location, since this location contains the target on the majority of trials. An improvement in performance due to reward-induced motivation that does not interact with cue validity may indicate a general alerting effect due to the reward.

The present study was designed to investigate the effect of reward-induced motivation on true exogenous attention orienting and IOR. We used a variant of the exogenous orienting task as used in Handy et al. (1999), in which attention was captured by a peripheral onset cue that did not predict the target location. Depending on their performance, participants had a chance on receiving low (i.e., € 0.10) or high (i.e., € 1.00) monetary reward in an alternating blocked design manner. In all cases, participants also received feedback about their performance. Short and long target–cue SOAs were varied within blocks.

Besides sustained block effects, we wanted to explore more transient effects of reward-induced motivation and interactions between motivational and cognitive control mechanisms. Since it is known that motivational and cognitive control functions are integrated (Kouneiher, Charron, & Koechlin, 2009), we hypothesized that under a high-reward-induced motivational state, cognitive control mechanisms are recruited to a greater extent. In order to test this, we investigated behavioral differences following trials that had a correct versus an incorrect response, since it is known that, following errors, an increase in cognitive control may improve the efficiency of information processing (Botvinick, Braver, Barch, Carter, & Cohen, 2001). Due to speed/accuracy trade-offs, this increase in efficiency is often expressed in “posterror-slowing,” the fact that reaction times (RTs) typically become longer after errors (Rabbit, 1966; Ridderinkhof, van den Wildenberg, Segalowitz, & Carter 2004b). Following our reasoning, there should be more posterror slowing in the high-reward, as compared with the low-reward, condition, because the high-reward condition evokes a high motivational state, which causes other top-down mechanisms, such as increased cognitive control, to be engaged more easily or more efficiently.

Furthermore, we wanted to determine whether reward-related personality differences were related to attention control. Therefore, we conducted the BIS/BAS questionnaire of Carver and White (1994). In Carver and White, a large-sample factor analysis identified a unidimensional BIS sensitivity measure and three BAS sensitivity subdimensions: BAS–Drive, BAS–Fun-seeking, and BAS–Reward responsiveness. The BIS and BAS scales reflect the sensitivity to the experience or anticipation of punishment and reward, respectively. The BAS–Drive scale measures the persistent pursuit of desired goals, the BAS–Fun-seeking subscale reflects both a desire for new rewards and a willingness to approach a potentially rewarding event on the spur of the moment, and the BAS–Reward responsiveness scale focuses on positive responses to the occurrence or anticipation of reward. The article wherein the BIS/BAS questionnaire was introduced (Carver & White, 1994) describes a reward study in which rewards could be obtained per block, similar to this study. The results showed that out of the three BAS subscales, the BAS–Drive subscale had the highest construct validity and is possibly the most important and useful scale for predicting reward-related behavior. More recent reward research has confirmed this by showing that out of all BIS/BAS scales, the BAS–Drive scale is the best predictor for reward-induced behavior (Hickey, Chelazzi, & Theeuwes, 2010b). Given that we were particularly interested in how trait propensity to reward-driven behavior is related to the way reward-induced motivation affects orienting and IOR, the BAS–Drive subscale was selected for further analysis.

In summary, the purpose of this study was threefold. First, we investigated sustained reward-induced motivational effects on orienting and reorienting of attention in a true exogenous cuing task. Second, we examined the transient effects of how the reward manipulation interacted with top-down cognitive control processes on a trial-by-trial basis. Third, we determined how personality trait propensity to reward-driven behavior was related to the way reward-induced motivation modulated attentional processes (e.g., orienting and reorienting) in our exogenous cuing task.

## Method

### Participants

Thirty-five students from the Vrije Universiteit Amsterdam participated in the experiment (16 males, 19–30 years of age, mean = 24.4 years, standard deviation = 2.7 years). All participants reported having normal or corrected-to-normal vision and gave written informed consent before participation. Participants received € 8.00 for participation and could earn between € 0.00 and € 8.80 extra reward depending on their performance. All research was approved by the Vrije Universiteit Faculty of Psychology ethics board and was conducted according to the principles of the Declaration of Helsinki.

### Stimuli and apparatus

An HP Compaq d530 CMT Pentium IV computer running OpenSesame (Mathôt, Schreij, & Theeuwes, 2012) generated the stimuli on a 22-in. Samsung Syncmaster 2233RZ screen (resolution 1,680 × 1,050, refreshing at 120 Hz) and acquired the necessary response data through the standard keyboard. All visual stimuli were presented on a gray (CIE: *x* = .068, *y* = .567; 0.71 cd/m^2^) background at a viewing distance of 70 cm. Auditory stimuli were presented through Sennheiser HD202 headphones. The sequence, timing, and characteristics of the stimuli were equal to those in Handy et al. (1999), although the spatial configuration of the display was adjusted to prevent anticipatory eye movements toward the possible target locations. In Handy et al. (1999), the two possible target locations were both in the upper half of the screen (one left and one right of the vertical meridian), so that an initial eye movement upward would be beneficial for detecting the target at both locations. Here, we prevented anticipatory eye movements by placing the target boxes left and right of the fixation cross on the horizontal meridian. At all times, a white (CIE: *x* = .255, *y* = .437; 67.11 cd/m^2^) fixation cross (0.33°) was displayed at the center of the screen. To indicate trial start, two gray (CIE: *x* = .298, *y* = .511; 12.12 cd/m^2^) square boxes (1.5°) appeared on the horizontal meridian 5° left and right from the fixation cross. A horizontally or vertically oriented yellow (CIE: *x* = .342, *y* = .576; 97.44 cd/m^2^) bar, 2 pixels wide and from 8 to 18 pixels long (adjustments in length by staircase procedure), served as the target stimulus and always appeared at the center of one of the two boxes. The nonfoveal cue was a brief brightening of the outline of one of the two boxes, and the central cue was a 0.33° filled white (CIE: *x* = .255, *y* = .437; 67.11 cd/m^2^) square presented on top of the fixation cross. The mask consisted of 16 (8 more horizontally and 8 more vertically oriented) yellow (CIE: *x* = .342, *y* = .576; 97.44 cd/m^2^) 2-pixels-wide bars that were randomly drawn through the box where the stimulus just had appeared. Visual feedback in the low-reward condition consisted of a single euro sign (i.e., “ €”), and feedback in the high-reward condition consisted of five euro signs (i.e., “€€€€€”). Feedback was presented 0.33° above the fixation cross for correct and incorrect responses in green (CIE: *x* = .220, *y* = .670; 69.97 cd/m^2^) and red (CIE: *x* = .593, *y* = .383; 24.86 cd/m^2^), respectively. Together with the visual feedback, auditory feedback was presented through headphones. In both the low- and high-reward conditions, auditory feedback consisted of a 200-ms pure tone with a frequency of 600 Hz for correct responses and 250 Hz for incorrect responses. During the intertrial interval, only the fixation cross remained present at the screen.

### Procedure 

**Fig. 1:**
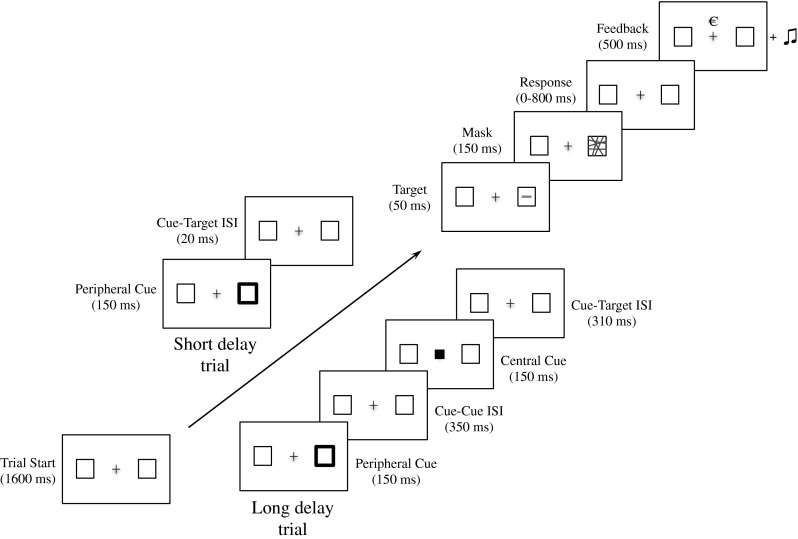
Sequence and timing of stimulus events in short (above arrow) and long (below arrow) delay trials. On every trial, the target stimulus was equally likely to be a horizontal or vertical bar presented on the left or right side of the fixation cross. All trials ended with a 400-ms intertrial interval in which only the fixation cross was displayed (not shown in figure). The proportions are not drawn to scale. ISI = interstimulus interval

The experiment took about 60 min. Participants were instructed to maintain fixation and make a discrimination judgment between a horizontally or vertically oriented bar that appeared at one of two possible peripheral stimulus locations (center of gray boxes) (see Fig. 1). It was emphasized that participants had to respond as quickly and accurately as possible. The experiment started with two practice blocks, followed by eight high-reward and eight low-reward blocks. High- and low-reward blocks were alternately presented, and the condition of the starting reward block (high or low) was counterbalanced across participants.

At the beginning of each block, participants were informed about the reward amount that they could win in that block. In high-reward blocks, participants had a 50 % chance of winning € 1.00, and in low-reward blocks, participants had a 50 % chance of winning € 0.10, if their performance was above threshold. The accuracy threshold was fixed for all participants at 68 % correct, and the RT threshold was individually set to the participant’s own average correct RT from the two practice blocks. After the practice blocks, participants were explicitly told about these thresholds and were instructed that the goal of the experiment was to win as much money as possible. At the end of each block, participants were informed about their performance and, if accuracy and RT were above and below threshold, respectively, advanced to a slot-machine-like lottery in which they had a 50 % chance of wining that block’s reward. If participants were not accurate or fast enough in a particular block, they did not advance to the lottery and were not able to win the reward of that block. Obtaining reward thus depended on a combination of chance and average block performance.

Each block consisted of 48 trials, which were equally split (but randomly varying) into validly versus invalidly cued and short versus long cue–target delay trials. The cues were nonpredictive for the location of the target bar (e.g., 50 % valid and 50 % invalid trials), and the orientation of the stimulus was equiprobable (50 % horizontally and 50 % vertically oriented bars). A valid trial was defined as a stimulus bar being presented at the cued peripheral location, and an invalid trial was defined as a stimulus bar being presented at the uncued (opposite to the cued location) peripheral location. To indicate the start of a trial, the two target boxes appeared next to the fixation cross for 1,600 ms. Thereafter, a peripheral cue was presented for 150 ms to the left or right of fixation to manipulate covert exogenous attention. On the short cue–target delay trials, the peripheral cue was followed by a cue–target delay of 20 ms, after which the stimulus was immediately presented for 50 ms. On the long cue–target delay trials, the peripheral cue was followed by a 350-ms cue–cue interval, after which the central cue appeared for 150 ms to return attention to fixation (as in Posner & Cohen, 1984). After this second cue, a cue–target interval of 310 ms preceded the stimulus presentation period of 50 ms. On all trials, the stimulus was immediately masked by presenting the mask at stimulus location for 150 ms. Within a 800-ms response window from mask disappearance, participants were required to report the stimulus bar’s orientation by pressing the “z” or the “m” keyboard button. Response buttons for vertically or horizontally oriented bars were counterbalanced across participants. Immediately following response, visual and auditory feedback were simultaneously delivered for 500 ms. All trials were separated by a fixed 400-ms intertrial interval. In the practice blocks, a staircase procedure for approximately 70 % correct performance was used in order to minimize the possibility of floor or ceiling effects in accuracy. For a total of 96 trials (48 trials × 2 practice blocks), performance was calculated every 8 trials. If accuracy was below 58 %, stimulus bar size was increased by 1 pixel to make the discrimination judgment easier. If accuracy was above 85 %, stimulus bar size was reduced by 1 pixel to make the discrimination judgment more difficult. Bar size started at 15 pixels (~0.35°) and could vary between 4 and 18 pixels. The bar could undergo a maximum of 12 size changes before it was set to a fixed size for all experimental trials. By using this procedure, we ensured that overall performance was approximately 70 % correct during the main experiment.

### Behavioral performance

All trials on which a response was made within the required response window (200–1,000 ms after stimulus onset) were categorized into the eight different conditions of the 2 × 2 × 2 design—that is, reward (high/low), delay (short/long), and validity (valid/invalid). A repeated measures analysis of variance (ANOVA) with reward (high/low), delay (short/long), and validity (valid/invalid) as factors was performed on the RT and accuracy measures to investigate sustained effects of reward on attention.

Besides sustained block effects, we explored more transient effects of reward-induced motivation and interactions between motivational and cognitive control mechanisms. A trial-by-trial analysis was performed investigating differences in RTs for trials following correct versus incorrect responses. Incorrect (after incorrect trials) and no-response trials were excluded, as well as the first trial of each block, since no response directly preceded these trials. The factor of previous trial (correct/incorrect) was added as another factor, and mean RTs were calculated per condition. A repeated measures ANOVA with reward (high/low), delay (short/long), validity (valid/invalid), and previous trial (correct/incorrect) as factors was performed.

### BIS/BAS personality inventory

After completion of the experimental task, participants filled in the BIS/BAS personality inventory (Carver & White, 1994) to measure trait propensity to reward-driven behavior. The BIS/BAS questionnaire consists of 24 Likert-type items (e.g. “When I want something I usually go all-out to get it”), with responses made on a 4-point response scale, with 1 indicating *strong agreement* and 4 indicating *strong disagreement* (with no neutral response). Here, we were interested only in the BAS–Drive subscale, which assesses trait propensity to reward-driven behavior.

In order to investigate whether personality was related to the way reward-induced motivation influences orienting and reorienting, two reward difference scores (i.e., the reward orienting score and the reward reorienting score) were calculated for both RT and accuracy measures. The reward orienting score was calculated by subtracting the cue facilitation effects (short-delay valid trials − short-delay invalid trials) from the high- and low-reward conditions. The reward reorienting score was calculated by subtracting the IOR effects (long-delay invalid trials − long-delay valid trials) from the high- and low-reward conditions.

The reward orienting and reorienting scores thus indicate to what degree a high-, as compared with a low-, reward-induced motivational state increases the cue facilitation and IOR effects, respectively. We correlated the individual BAS–Drive scale scores and the reward orienting and reorienting scores for both RT and accuracy to investigate whether trait propensity to reward-driven behavior was related to changes in orienting and reorienting behavior in our reward task.

## Results

Participants earned between € 0.00 and € 6.20 extra reward (mean € 2.35, standard deviation € 1.65), and target length was between 6 and 15 pixels (mean 11 pixels, standard deviation 3 pixels). The accuracy scores of 5 participants were below the 50 % chance level in one or more conditions, and therefore, their data were excluded from analyses.

### Attentional facilitation and inhibition

Mean RTs and accuracies are presented in Table 1. A repeated measures ANOVA on mean RT with reward (high/low), delay (short/long), and validity (valid/invalid) as factors showed a significant main effect of delay, *F*(1, 29) = 101.09, *p* < .001, and of validity, *F*(1, 29) = 5.79, *p* < .05. Crucially, there was a significant interaction between delay and validity, *F*(1, 29) = 19.61, *p* < .001, suggesting that cue validity had a different effect in the short-delay, as compared with the long-delay, condition, a result typically found in tasks investigating cue facilitation effects and IOR.

**Table 1:** Mean (standard deviation) reaction times and accuracy across participants as a function of cue–target delay and cue validity separately presented for the low- and high-reward conditions

	Low reward		High reward	
	Valid cue	Invalid cue	Valid cue	Invalid cue
Delay	Reaction time (ms)			
Short	523 (69)	541 (74)	519 (66)	540 (78)
Long	494 (74)	496 (86)	495(79)	484 (82)
	Accuracy (% correct)			
Short	73.7 (9.9)	67.7 (8.9)	74.8 (11.1)	70.4 (8.3)
Long	72.8 (9.0)	74.7 (9.9)	74.2 (8.6)	76.8 (8.6)

**Fig. 2:**
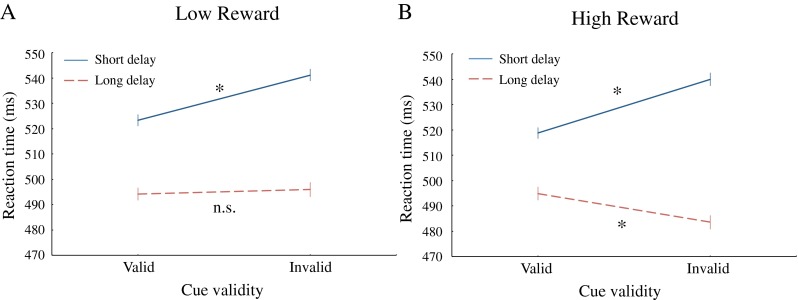
Three-way interaction effect between reward, delay, and validity plotted separately for the low-reward (**a**) and the high-reward (**b**) conditions. Error bars represent standard errors of the means

Moreover, a significant three-way interaction between reward, delay, and validity, *F*(1, 29) = 6.70, *p* < .05, was observed. Figure 2 displays this interaction and reveals that in the short cue–target delay condition, cue validity had the same effect in the low- and high-reward conditions, *t*(29) < 1, suggesting that initial attentional orienting toward the cue was not affected by reward-induced motivation. Crucially, however, in the long-delay condition, a different pattern of results was found: In the high-reward condition, the classic IOR effect was found, with slower responding in the valid versus the invalid cue condition, while in the low-reward condition, there was no effect of cue validity.

Subsequent two-tailed *t*-tests confirmed this pattern of results, with faster responses for valid versus invalid cues after short cue–target delays, for both the low-reward, *t*(29) = 3.85, *SE* = 4.65, *p* < .001, and the high-reward, *t*(29) = 4.42, *SE* = 4.80, *p* < .001, conditions. However, IOR was found only in the high-reward condition, *t*(29) = 2.80, *SE* = 4.01, *p* < .01, and not in the low-reward condition, *t*(29) < 1. These results indicate that reward had basically no effect on initial orienting of attention, since the cuing effects in the short-delay condition were not modulated by the reward. However, in the low-reward condition, initial facilitation was not followed by IOR, while this was the case in the high-reward condition. Clearly, reward-induced motivation had an effect on reorienting and the accompanying inhibitory effects that followed the initial capture of attention by the abrupt onset cues.

A repeated measures ANOVA on mean accuracy with reward (high/low), delay (short/long), and validity (valid/invalid) as factors showed a significant main effect of reward, *F*(1, 29) = 5.10, *p* < .05, suggesting that participants performed more accurately in the high- than in the low-reward condition. Additionally, a main effect of delay, *F*(1, 29) = 10.03, *p* < .01, and of validity, *F*(1, 29) = 7.15, *p* < .05, was observed, with more accurate responses on long-delay trials and validly cued trials. Furthermore, there was a significant interaction between delay and validity, *F*(1, 29) = 33.97, *p* < .001, following the pattern typically found in tasks investigating cue facilitation effects and IOR.

### Transient effects of reward

**Fig. 3:**
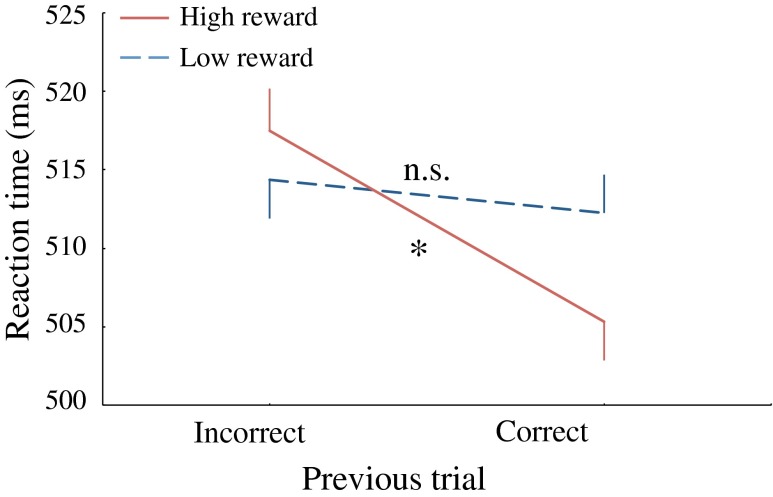
Mean reaction times for low- and high-reward condition trials following an incorrect or correct trial. Note that posterror slowing is observed only in the high-reward condition. Error bars represent standard errors of the means

To explore the more transient effects of reward-induced motivation and interactions between motivation and cognitive control, we conducted a trial-by-trial analysis determining whether errors were followed by posterror slowing. A repeated measures ANOVA on mean RT with reward (high/low), delay (short/long), validity (valid/invalid), and previous trial (correct/incorrect) as factors, showed a significant main effect of previous trial, *F*(1, 29) = 5.41, *p* < .05, with slower responses for trials following an error. Yet crucially for the present analysis, there was a significant interaction effect between reward (high/low) and previous trial (correct/incorrect), *F*(1, 29) = 4.76, *p* < .05, suggesting a different effect of high- versus low-reward-induced motivation on posterror responding. Subsequent two-tailed *t*-tests showed no difference in RT following correct or incorrect responses in the low-reward condition, *t*(29) < 1, but significant posterror slowing in the high-reward condition, *t*(29) = 3.17, *SE* = 3.86, *p* < .01. As is clear from Fig. 3, making an error slowed the response significantly, relatively to when no error was made, in the high-reward condition, but not in the low-reward condition. Furthermore, there was no interaction between previous trial (correct/incorrect) and delay (short/long) or between previous trial (correct/incorrect) and validity (valid/invalid).

### Reward effects and personality

The BIS/BAS personality inventory was assessed in order to measure trait propensity to reward-driven behavior. The BIS/BAS inventory results were consistent with those reported in earlier studies (Carver & White, 1994; Hickey et al., 2010b), with mean (standard deviation) for BIS 18.8 (3.6), BAS–Overall 13.9 (1.5), BAS–Drive 11.8 (2.1), BAS–Fun-seeking12.3 (2.1), and BAS–Reward responsiveness 17.6 (1.9). The BIS and BAS, which are theoretically orthogonal constructs (Gray, 1987), did not show a reliable relationship (*ρ* = −.025), and the BAS subscales showed a stronger relationship (*ρ* between .276 and .325). Note that correlation values reflect Spearman’s *ρ*, which is less sensitive to outliers, as compared with other measures of correlation.

To investigate whether personality was related to the way reward-induced motivation influences orienting and reorienting, we correlated the individual BAS–Drive scale scores and the reward orienting and reorienting scores (see Table 2). The reward orienting and reorienting scores reflect to what degree a high-reward-induced, as compared with a low-reward-induced, motivational state increases the cue facilitation and IOR effects, respectively. A higher BAS–Drive scale indicates a higher tendency to reward-driven behavior. We observed a significant positive correlation between the reward reorienting score on accuracy and the BAS–Drive score (see Table 2). This implies that participants who show a larger tendency toward reward-driven behavior show a larger change in reorienting behavior (larger IOR effect) due to reward. The absence of such a significant correlation with the reward orienting scores suggests that reward-related personality traits can be linked to reorienting, but not orienting, behavior.

**Table 2:** Correlations between the individual BAS-Drive scale scores and the reward orienting and reorienting scores for both reaction times and accuracy

	Reaction time orienting	Reaction time reorienting	Accuracy orienting	Accuracy reorienting
BAS-Drive				
Spearman’s *ρ*	.154	−.131	−.283	.394*
*p*-value	.416	.492	.129	.031

*Significant correlation

## Discussion

In the present study, we investigated the effect of reward-induced motivation on exogenous attention orienting and IOR. Typical cue facilitation effects on initial orienting were observed for both the low- and high-reward conditions. However, IOR was found only for the high-reward condition, indicating that reward-induced motivation had an effect on inhibitory processes that followed the initial capture of attention.

Consistent with other studies manipulating reward-induced motivation, we found an enhancement of behavioral performance in high-reward, relative to low-reward, blocks (Engelmann & Pessoa, 2007; Small et al., 2005). Unlike these previous studies (Engelmann & Pessoa, 2007; Small et al., 2005), we did find direct evidence that the reorienting of attention is affected by reward. Crucially, however, we showed that the initial orienting of attention is not affected by reward, since in the short-delay condition, the difference between validly and invalidly cued conditions was similar for high- and low-reward conditions (see Fig. 2). If initial orienting of attention to the cued location had been quicker in the high-reward than in the low-reward, condition, one would have expected better performance for targets at the validly cued location in the high-reward condition. This was clearly not the case (see Table 1), which indicates that cue facilitation effects on initial orienting are not modulated by the manipulation of reward-induced motivation.

However, there was an effect of reward-induced motivation on the reorienting of attention and the accompanying inhibitory processes following initial orientation. In the high-reward condition, participants were quicker to reorient attention away from the initially cued location, since they were faster for targets in the invalidly cued condition, relative to the validly cued condition (the well-known IOR effect). In the low-reward condition, this effect was basically not present (valid and invalid conditions gave the same performance), suggesting that observers did not necessarily reorient away from the cued location. The latter finding is important because it suggests that a process such as IOR, which has always been considered to be very much stimulus-driven, is affected by reward-induced motivational factors.

With regard to the initial orienting of attention, our results are consistent with those of previous studies investigating orienting (Engelmann & Pessoa, 2007; Small et al., 2005), since in these previous studies, reward-induced motivation did not modulate cue validity effects. This finding is also consistent with that of Shomstein and Johnson (2013), who showed that space-based (and not object-based) guidance of attention is robust to reward influences. Under low- and high-reward conditions, the reward manipulation did not modulate the effect of initial orienting to the location that was indicated by the abrupt onset cue. Importantly, Shomstein and Johnson also showed that object-based attention, which is often also considered to be automatic and stimulus-driven, was in fact very much affected by a reward-based strategy. Observers were more likely to direct attention to the “different” object (instead of the typical same-object advantage seen in object-based experiments) when the reward contingencies were biased toward a different-object advantage. Using the same paradigm, a functional magnetic resonance imaging study by Lee and Shomstein (2013) provided neural evidence supporting the above-mentioned findings. Immediately after cue onset, retinotopic areas V1–V4 mandatorily showed an automatic space-based effect that was not influenced by the presence of a reward-based strategy. However, the space-based related activity in retinotopic areas disappeared over time, indicating that with sufficient time, spatial orienting was modulated by a reward-based strategy. All these findings, including our own, converge on the same conclusions. Immediate spatial exogenous attention is fully automatic, is stimulus-driven, and is not modulated by motivational context (Milliken, Tipper, Houghton, & Lupiáñez, 2000; Posner, Snyder, & Davidson, 1980; Schreij, Owens, & Theeuwes, 2008; Theeuwes, 1994). However, with sufficient time, reward-induced motivation can affect attentional processes (Lee & Shomstein, 2013; Shomstein & Johnson, 2013), including the IOR effect.

The present results, then, call into question the notion that IOR is only automatic and completely stimulus-driven. If reward contingencies are able to affect IOR, it should be modulated by top-down mechanisms. Consistent with this claim, some earlier studies demonstrated that volitional control can affect IOR (e.g., Jones, Moynihan, MacKenzie, & Puente, 2002; Khatoon, Briand, & Sereno, 2002; Lupiáñez & Milliken, 1999; Lupiáñez, Milliken, Solano, Weaver, & Tipper 2001; Tipper & Kingstone, 2005). For example, Tipper and Kingstone manipulated the number of catch trials (trials on which the cue was not followed by the target) so that the reliability of peripheral cues as temporal warning signals varied between conditions. Their results showed that when a peripheral cue was unreliable (i.e., high number of catch trials) and not used as a warning signal, the IOR effect was reduced significantly. This suggests that, depending on the beliefs or mental state of the observer, the IOR effect can be modulated by volitional top-down control processes. Similarly in our study, we assume that the mental state of participants differed between the high- and the low-reward conditions in such a way that motivational top-down processes were engaged differently. Under the condition of high-reward-induced, as compared to with low reward-induced, motivation, volitional top-down control processes were thought to be employed to a greater extent, which resulted in modulation of the IOR effect.

Importantly, we show that in a pure exogenously (nonpredictive cues) Posner-type cuing task, initial orienting is not modulated by reward-induced motivation. However, the inhibitory processes following initial orientation are modulated by reward-induced motivation. When the reward-induced motivational state is low, initial cue facilitation is not followed by inhibition of that location, while facilitation is followed by inhibition of the initially cued location when the reward-induced motivational state is high. This suggests that reward-induced motivation interacts with reorientation processes that are accompanied by IOR, but not with initial orientation toward the cue. Although Engelmann and Pessoa (2007) concluded differently, they acknowledged the possibility that reward information affects orientation and reorientation processes differently. Lacking a significant interaction between validity and reward incentive, they concluded that increased motivation, induced by an increase in monetary reward, enhanced both orienting and reorienting performance. As was noted, it could be that the observed overall performance increase in their study was due to a general alerting effect.

Although reward-induced motivation was not able to influence initial stimulus-driven orientation, we suggest that IOR, which occurs later in time, can be influenced in a top-down manner. This dissociation can be explained by the fact that two different attentional systems, with distinct underlying neural networks (Fecteau & Munoz 2005; Kincade, Abrams, Astafiev, Shulman, & Corbetta, 2005), are involved in orienting to a cued location and disengaging from a cued location to enable reorienting (Corbetta & Shulman, 2002). While validly cued targets evoke a single attention-guiding process, mediated by the orienting network (Thiel, Zilles, & Fink, 2004), invalidly cued targets evoke several processes, including disengagement from the cued location and shifting attention to another location, mediated by the reorienting network (Corbetta, Kincade, Ollinger, McAvoy, & Shulman, 2000). It is known that the latter reorientation network can be influenced by properties that make target stimuli more salient (Downar, Crawley, Mikulis, & Davis, 2002), including increased reward magnitude (Engelmann & Pessoa, 2007). We suggest that the reorientation of attention and the accompanying IOR effect are in themselves not completely stimulus-driven and partially involve top-down processes. Therefore, it is possible for motivational top-down processes, such as those induced by reward, to modulate reorienting and IOR.

To support this hypothesis of co-acting top-down mechanisms, we performed a trial-by-trial analysis investigating the interaction between reward-induced motivation and top-down cognitive control. In order to do this, we examined differences in performance following a correct versus an incorrect response, since it is well-know that cognitive control mechanisms are recruited to a greater extent after erroneous responses (Botvinick et al., 2001; Ridderinkhof, Ullsperger, et al., 2004; Ridderinkhof, van den Wildenberg, Segalowitz, & Carter, 2004). The increase in cognitive control improves the efficiency of information processing, which is, due to speed/accuracy trade-offs, often expressed in posterror slowing (Rabbit, 1966). Similar to earlier studies showing an increase in top-down control under high motivational states (Jimura et al., 2010, Padmala & Pessoa, 2011), we found that in the high-reward, as compared with the low-reward, condition, participants exerted more cognitive control after erroneous responses, exposed by an increase in posterror RTs. We hypothesize that the high-reward condition evokes a high motivational state, which causes other top-down mechanisms, such as cognitive control, to be engaged more easily or more efficiently. While it is known that motivational and cognitive control functions are integrated to serve decision making (Kouneiher et al., 2009), Locke and Braver (2008) showed that, depending on different reward and punishment conditions, participants can even alter their cognitive control strategy differently, suggesting that top-down processes can interact in a specific way to enhance behavioral performance. Their neuroimaging data showed that the reward condition was associated with activation in a mainly right-lateralized network including areas that are linked to exogenous attention and IOR (e.g., regions around the temporo-parietal junction and inferior frontal gyrus; Corbetta & Shulman, 2002). These functional-anatomical similarities are consistent with the proposed hypothesis of co-acting top-down processes in our cuing task. Moreover, Locke and Braver found that individual differences in motivation-induced performance enhancement through increased cognitive control were linked to the BAS scale of the BIS/BAS personality inventory that was also conducted in our study.

Here, we were specifically interested in the BAS–Drive scale, which is suggested to be the best predictor of behavior in blocked reward tasks (Carver & White, 1994). The BAS–Drive scale measures trait propensity to reward-driven behavior, with higher BAS–Drive scales indicating a higher tendency to reward-driven behavior. We correlated the BAS–Drive scale with the reward orienting and reorienting scores. The reward orienting and reorienting scores, respectively, reflect to what degree a high-reward-induced, as compared with a low-reward-induced, motivational state increases the cue facilitation effect and the IOR effect. The analysis showed a significant positive correlation of the BAS–Drive score with the reward reorienting score on accuracy. This implies that participants with a higher tendency toward reward-driven behavior, due to a larger difference in motivational states between low- and high-reward blocks, show a larger difference in reorienting performance between the reward conditions, with an increased IOR effect in the high-reward, as compared with the low-reward, condition. Crucially, the absence of such a significant correlation with the reward orienting scores, suggests that reward-related personality traits influence reorienting, and not orienting, behavior. This result is consistent with the RT data that show that reorienting, and not orienting, is influenced by reward-induced motivation.

These results are consistent with those of Locke and Braver (2008), who showed a direct relation between the cumulative BAS scale and motivation-induced performance enhancement through increased cognitive control. In high-reward, as compared with low-reward, blocks, we observed an advantageous change in reorienting behavior (IOR) that was accompanied by an increase in top-down cognitive control, as well as individual differences in reward reorienting behavior (reward reorienting scores) that were linked to the BAS–Drive scale. Together, these findings indicate that personality trait propensity to reward-driven behavior is related to performance enhancement under high-reward-induced motivational states, during which there is an increase in top-down cognitive control. Although the exact relationship between reward-related personality traits and top-down processes still needs to be disclosed, these results permit one to suggest that reward-induced motivational processes and cognitive control processes co-act in order to improve behavioral performance.

In conclusion, the present study shows that reorienting, and not initial orienting, is affected by reward-induced motivation. Typical cue facilitation effects on initial orienting did not differ between the low- and high-reward conditions. However, reward-induced motivation had a clear effect on inhibitory processes following the initial capture of attention, since we found IOR in high- but not in low-reward blocks. Moreover, we revealed that motivation-induced behavioral enhancement was associated with increased cognitive control, which may be affected by the way individuals show tendency to reward-driven behavior.
